# Relationship between myopia control and amount of corneal refractive change after orthokeratology lens treatment

**DOI:** 10.1186/s12886-023-03178-8

**Published:** 2023-10-30

**Authors:** Lu Sun, Hong-Xin Song, Zheng-Xuan Li, Yun Chen, Zhi-Qiang He

**Affiliations:** 1 Beijing Aier-Intech Eye Hospital, Beijing, 100021 China; 2 Beijing Tongren Eye Center, Beijing Institute of Ophthalmology, Beijing Tongren Hospital, Capital Medical University, Beijing Key Laboratory of Ophthalmology and Visual Sciences, National Engineering Research Center for Ophthalmology, #1 Dong Jiao Min Xiang, Beijing, 100730 China; 3 Key Laboratory of Universal Wireless Communications, Ministry of Education, Beijing University of Posts and Telecommunications, No. 10 Xitucheng Road, Beijing, 100876 China

**Keywords:** Orthokeratology, Corneal refractive change region, Axial length, Myopia control

## Abstract

**Background:**

To evaluate the relationship between amount of corneal refractive change (CRC) after wearing orthokeratology (Ortho-K) lenses and axial length (AL) growth.

**Methods:**

We retrospectively enrolled 77 patients (77 eyes) aged 8–14 years who wore Ortho-K lenses more than 12 months. We divided the patients into 2 subgroups: spherical equivalent (SE) ≤ -3.0 D and SE > -3.0 D subgroup. The sagittal and tangential curvature maps and corneal topographic data within the 8-mm diameter ring at the baseline and during follow-up visits after wearing Ortho-K lens were recorded in addition to the area, height, and volume of the CRC region. The AL data were recorded at the baseline and during follow-up visits. Multivariate linear regression was conducted to analyze associations between the area, height, and volume of the CRC region, AL elongation, and SE.

**Results:**

The average change in the CRC region was 9.77 ± 0.60 D in height, 16.66 ± 3.61 mm^2^ in area, and 87.47 ± 8.96 D*mm^2^ in volume on the tangential diagram after wearing Ortho-K lenses for 3 months. The AL showed a change of 0.19 ± 0.14 mm after 1 year of Ortho-K lens wear (*P* < 0.05). At 1 year, AL elongation was negatively correlated with the area (*P* = 0.019) and volume (*P* < 0.001) of the CRC region. At 1 year, for every 1-mm^2^ increase in the area and every 1-D*mm^2^ increase in the volume of the CRC region, the average AL elongation decreased by 0.01 mm and 0.002 mm, respectively, in the multivariate analysis. In patients with SE ≤ -3.0 D, AL elongation was negatively correlated with the CRC-region volume (β = -0.002, P = 0.018), and in patients with SE > -3.0 D, AL elongation was negatively correlated with the CRC-region area (β = -0.017, *P* = 0.016).

**Conclusions:**

The AL elongation-control efficacy of Ortho-K lenses may be related to the area and volume of the CRC region.

**Supplementary Information:**

The online version contains supplementary material available at 10.1186/s12886-023-03178-8.

## Background

Myopia has become a major public health issue, as its prevalence has been increasing in the last few decades. It is estimated that by 2050, around 5 billion people will be myopic [1]. Importantly, children who develop myopia at an early age are more likely to experience severe myopia-related complications later in life, such as glaucoma, chorioretinal degeneration, and retinal detachment [2, 3]. The risk of these complications increases with the severity of myopia and the elongation of axial length (AL) [4]. Therefore, the prevention of myopia onset and inhibition of myopia progression are of great clinical significance.

Although the exact pathogenesis of myopia remains controversial, an animal experiment has confirmed that stimulation by hyperopic defocus on the retina caused by a negative lens can accelerate AL elongation [5]. Moreover, recent studies [6, 7] have shown that in addition to the central retina, the peripheral retina also affects refractive development in animals. These findings have significant clinical implications, because studies involving human subjects have indicated that intrinsic peripheral hyperopic defocus may be related to the early onset of myopia in children [8]. Various optical devices have been used in clinics to slow down the progression of myopia. For example, orthokeratology (Ortho-K) lenses are rigid gas-permeable lenses that are specially designed to change the curvature of the anterior surface of the cornea. Ortho-K lenses are worn at night, and the resulting change in the curvature of the anterior corneal surface after their removal results in correction of the refractive error [9]. Flattening of the central cornea can correct axial myopia, while steepening of the mid-peripheral cornea can reduce peripheral hyperopia [10]. Both changes can increase the peripheral myopic defocus region, which can reduce the hyperopic defocus on the peripheral retina, thus reducing the visual feedback for AL elongation and retarding myopia progression [11–13].

According to Wallman and Winawer [14], vision is the main input stimulus for the guidance of ocular growth. Although the visual signal conveying size information is still unknown, studies have shown that the eye can encode size from defocusing the image. Ortho-K lenses change the peripheral optics of the eye by shifting the paraxial focus relative to the peripheral image and moving the peripheral image forward, resulting in relative peripheral myopic defocus [15, 16].

The myopia-control effect of Ortho-K lenses varies among individuals, possibly due to differences in corneal surface refractive chang resulting from changes in the corneal area of visual signal transduction caused by the change in corneal refractive power induced by Ortho-K lenses. Studies have suggested that the greater the myopic defocusing induced by Ortho-K lenses, the greater is the impact on eye growth [17]. Hence, the myopia-control effect of Ortho-K lenses may contribute to changes in the corneal contour as observed on corneal topography [18]. Indeed, studies have reported that the morphological changes in corneal refractive power after wearing Ortho-K lenses are correlated with AL elongation, and measurement indices related to refractive power changes have been established [19–21]. These indices could be used to estimate and predict the myopia-control effect of Ortho-K lenses based on the refractive power change.

The above studies have suggested that the myopia-control effect of Ortho-K lenses is associated with changes in corneal refractive power, which can induce peripheral myopic defocus and ultimately reduce the visual feedback for AL elongation. However, a quantitative relationship between refractive power changes and defocusing has not been established. Hu et al. [22] created a model of corneal power shifts induced by Ortho-K lenses, and reported that the summed corneal power shift is negatively correlated with the AL elongation-control effect. The myopia-control effect is closely related to personal eye-care habits, such as outdoor activities, intensity of near and distance work, and other behavioral factors. In the present study, we focused on the relationship between myopia control and the CRC region induced by Ortho-K lenses. We used mathematical software to quantify CRC region to observe its relationship with the myopia-control effect. Our aim was to scientifically and objectively explain the existing phenomena provide evidence for the myopic defocus theory.

## Methods

### Subjects

We retrospectively analyzed data from 77 myopic children (77 eyes) who were treated with Ortho-K lenses for more than 12 months in the Beijing Tongren Hospital myopia-control outpatient clinic between June 2019 and September 2021. The inclusion criteria were as follows: age between 8 and 14 years, cycloplegic spherical power between − 0.75 diopters (D) and − 5.50 D, astigmatism between 0 D and − 1.50 D, and corrected distance visual acuity equal to or better than 20/20. The exclusion criteria were as follows: history of using orthokeratology lenses or other contact lenses or spectacles; history of other ocular diseases, such as congenital cataract, glaucoma, and strabismus; genetic history of high myopia; discontinuation of Ortho-K lens use during the study period (defined as not wearing the lenses for > 30 continuous days); and active inflammatory ocular-surface diseases. To eliminate between-eye correlations, we only analyzed the data of the right eye. The participants were scheduled to visit the clinic at the baseline and 1 day, 1 week, 1 month, 3 months, 6 months, 9 months, and 12 months after Ortho-K lens use. At each visit, all subjects underwent slit-lamp microscopy, refraction, visual acuity, corneal topography, and ocular biometry examinations.

This study adhered to the tenets of the Declaration of Helsinki. The study was reviewed by the ethics committee of Beijing Tongren Hospital, and conformed with the principles and applicable guidelines for the protection of human subjects in biomedical research.

### Lens fitting

The Ortho-K lenses used in the current study were either spherical 4-zone lenses (α ORTHO®-K, Menicon Co., Selangor, Japan) or 3-zone lenses (Paragon CRT™, Paragon Vision Sciences, Gilbert, AZ). To select the trial lens, we used flat keratometry and corneal eccentricity to determine the alignment curve radius. We used fluorescein staining to observe the central position of the Ortho-K lens: good centration was defined as a 0.5–1.0 mm range of movement after blinking with appropriate tightness and a 1.0–2.0 mm annular layer of tears trapped in the reverse curve, forming a bull’s eye configuration. Then, a properly fitting lens was chosen based on the fluorescein pattern observed under the slit-lamp microscope and corneal topography. Customized Ortho-K lenses were ordered, and the children were instructed to wear their lenses every night for at least 8 consecutive hours.

### Measurements

Cycloplegic refraction was performed at the baseline. Cycloplegia was achieved with 3 drops of 0.5% tropicamide at 5-min intervals.

At 10 min after the application of the third drop, autorefraction was performed 3 times (TOPCON, Japan, model: KR- 8100), and subjective refraction was performed after the autorefraction. The spherical equivalent (SE) was calculated as the spherical power plus 1/2 cylindrical power.

A noncontact partial coherence interferometer (IOL-Master; Carl Zeiss, Germany) was used to measure the AL at the baseline and at 6 and 12 months after orthokeratology lens wear. At each visit, the AL was measured 5 consecutive times, and the mean value was calculated.

Corneal topography (Medmont E300, Medmont International Pty Ltd., Australia) was used to collect corneal-surface data. Tangential power maps on corneal topography after 3 months of Ortho-K lens wear were used to quantify the total area, height, and volume of the CRC region. Since corneal reshaping by Ortho-K lenses is complete and stable at 7–10 days after wearing the contact lenses [23], the amount of CRC was calculated based on the corneal topography maps taken at the baseline and after 3 months of Ortho-K lens wear. The corneal parameters, including the baseline eccentricity (E) value, flat K value, steep K value, corneal curvature, and astigmatism, were obtained by a professional technician, and the best image was selected for analysis.

### Corneal defocusing

#### Acquisition of raw data

Data files (.*dst* files and .*tgl* files) were exported from Medmont E300 topographer. The .*dst* files recorded the radial distance between the collection points and the center of the cornea as $R=\{r_{i,j}\vert i=\mathit1\mathit,\mathit2\mathit,\mathit.\mathit.\mathit.\mathit,\mathit{300}\mathit,j\mathit=\mathit1\mathit,\mathit2\mathit,\mathit.\mathit.\mathit.\mathit,\mathit{32}\}$, and the .*tgl* files recorded the tangential curvature data of the cornea as $C\;=\;\left\{c_{i,j}\;\left|\;i\;=\;1,2,...,300,\;j=1,2,...32\right.\right\}$. Ideally, a total of 9,600 data points (300 × 32) would have been collected. However, due to various unavoidable reasons, there was some partial loss of data, resulting in a smaller number of data points collected. In cases where data was missing, a value of 0 was used to fill in the gaps.

#### Data preprocessing

The data were preprocessed to fit the missing data. For the radial data, linear interpolation was used to fit data with a value of 0. The tangential curvature data were transformed into tangential refractive power (in diopters) as follows: $D=\{ {d_{i,j}}|i=1,2,...,300,j=1,2,...,32|$.

The transformation is shown below:

$${d}_{i,j}=\frac{337.5}{{c}_{i,j}},i=\text{1,2},\dots ,300,j=\text{1,2},\dots ,32.$$

Then, any missing data with a value of 0 was also fitted using linear interpolation.

#### Mean filtering and smoothing

Mean filtering was used to remove noise and high-frequency information generated during the acquisition process, resulting in smoother data.

#### 3D modeling

According to the acquisition principle of Placido, 32 data points were collected at 1.2° intervals. This allowed for the location of each collection point on the cornea to be determined using the polar coordinate system. Additionally, each collection point corresponded to a tangential refractive power. When using the corneal center as the coordinate origin of the polar coordinate system, the coordinates of the collected corneal data points were as follows:

$$\begin{aligned} P&=\{\ \quad{p_{i,j}}\quad\ \ |i=1,2,\ldots,300,j=1,2,\ldots,32\} \hfill \\ &=\{ ({r_{i,j}},1.2i)|i=1,2,\ldots,300,j=1,2,\ldots,32\} \hfill \\ \end{aligned}$$

After transforming the polar coordinate system into a Cartesian coordinate system, the coordinates of the corneal data points were obtained as follows:

$$\begin{gathered} P=\{ {p_{i,j}}|i=1,2,\ldots,300,j=1,2,\ldots,32\} \hfill \\ =\{ ({r_{i,j}} \times \cos \theta ,{r_{i,j}} \times \sin \theta )|\theta =\frac{{1.2\pi i}}{{180}},i=1,2,\ldots,300,j=1,2,\ldots,32\} \hfill \\ \end{gathered}$$

Each coordinate ${p_{i,j}}$ corresponded to a diopter ${d_{i,j}}$, and the following three-dimensional coordinate point set was obtained:

$$\{ ({x_{i,j}},{y_{i,j}},{z_{i,j}})|i=1,2,\ldots,300,j=1,2,\ldots,32\}$$

in which, $${x}_{i,j}={r}_{i,j}\times {cos}\theta ,{y}_{i,j}={r}_{i,j}\times {sin}\theta ,{z}_{i,j}={d}_{i,j},\theta =\frac{1.2\pi i}{180}$$.

**Fig. 1:**
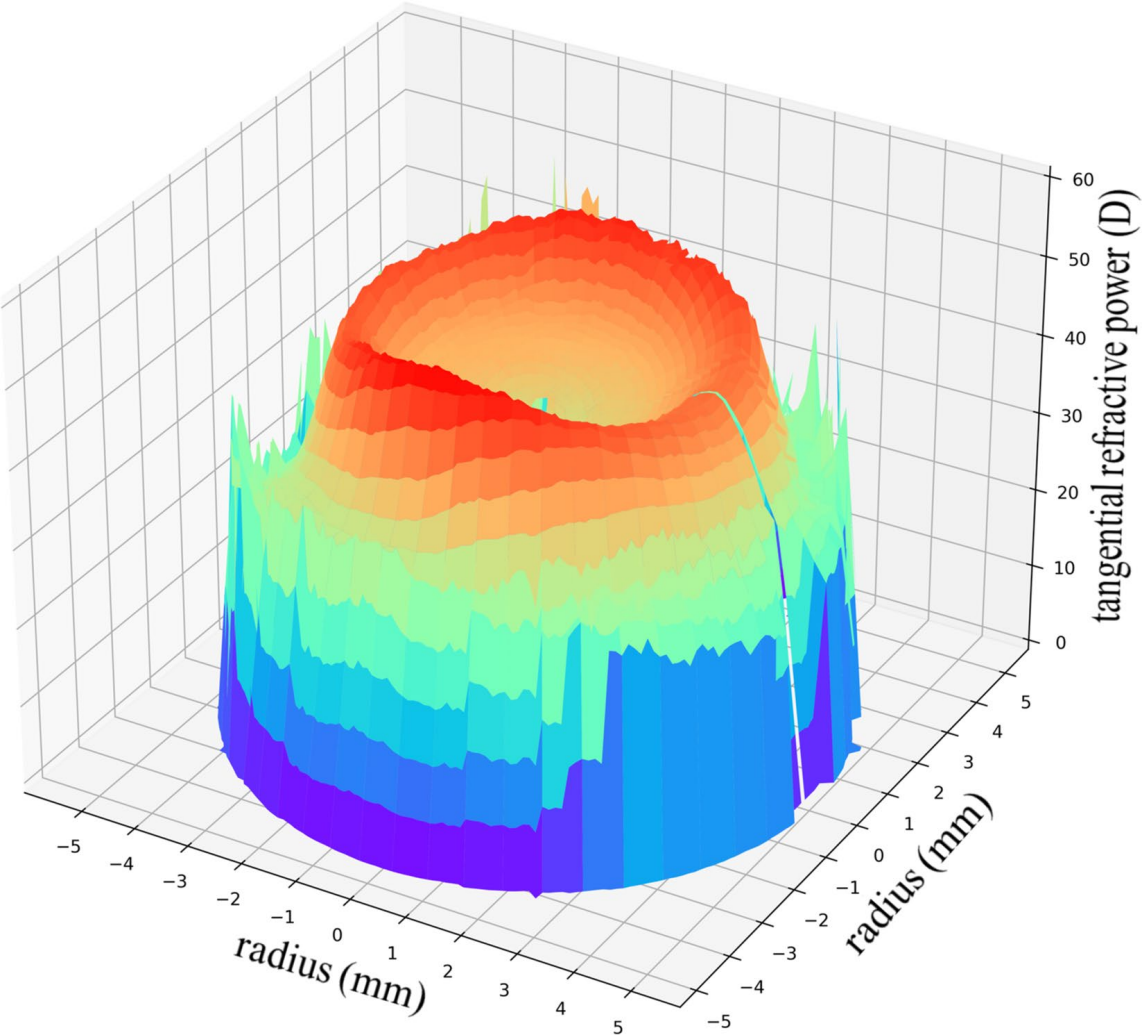
3D modeling of the corneal refractive change. Based on the obtained coordinate point set, 3D modeling was carried out by interpolation

Based on the obtained coordinate point set, 3D modeling was carried out by interpolation, and the results are shown in Fig. 1.

#### Polynomial fitting

The data was collected using the Medmont E300 topographer, which gathered 32 points at 1.2° intervals. In total, data points were collected in 300 directions. Subsequently, 64 points were selected by choosing those with an angle difference of 180°, and they were plotted on the Cartesian coordinate system.

A two-dimensional coordinate system was established by plotting the radial data on the horizontal axis and the tangential refractive power on the vertical axis, with the corneal center as the origin. Polynomial fitting was carried out for the 64-point scatter diagrams with the fitting formula shown below.

$${\text{y}}=f(x)=\sum\limits_{{i=0}}^{n} {{a_i}{x^i}}$$

in which,$n$ represents the highest degree of the polynomial; its value in this algorithm was 21. The fitting results are shown in Figure S1 (a).

#### Calculation of the corneal refractive change region

The CRC region was calculated based on a cross-section obtained in the following manner (Figure S1): First, the maximum points were calculated with the fitting function. The two maximum points with the highest values, located on opposite sides of the origin, were identified as the peaks. The minimum points between the two peaks were then determined, and the minimum points closest to the peaks were used to determine the boundary of the CRC region.

The mean CRC of all points between the two minimum points was calculated and used to determine the boundary of the CRC region. The corresponding radial distance was calculated based on the mean tangential refractive power. The results are shown in Figure S1 (b).

The difference between the peak and the mean tangential refractive power was defined as the height of this direction. The average of all the heights in all directions was considered as the height of the eye.

#### Calculation of relevant data

After obtaining the CRC region boundary on a straight line in step 6, four coordinate points were obtained, representing two radial distance coordinate points from the CRC region boundary in each of the two opposite directions. To ensure accurate calculations, these two coordinate points were replaced by the nearest two points obtained from the E300: ${d_{i,in}},{d_{i,out}}$. Two boundaries were calculated in each direction: the inner and outer radii of the CRC region. There were a total of 300 directions. Therefore, we calculated the area of the refractive change region of the entire eye. As shown in Figure S2, the red region is the CRC region. The volume of the CRC region could be calculated using the following formulae:

$$Are{a_{rc}}=\sum\limits_{{i=1}}^{{300}} {\sum\limits_{{j=in}}^{{out - 1}} {\frac{{1.2}}{{360}} \times \pi \times (r_{{i,j+1}}^{2}} - r_{{i,j}}^{2})}$$

$$Volum{e_{after}}=\sum\limits_{{i=1}}^{{300}} {\sum\limits_{{j=in}}^{{out - 1}} {\frac{{1.2}}{{360}} \times \pi \times (r_{{i,j+1}}^{2}} - r_{{i,j}}^{2})} \times \frac{1}{2} \times ({d_{i,j+1}}+{d_{i,j}})$$

$$Volum{e_{before}}=\sum\limits_{{i=1}}^{{300}} {\sum\limits_{{j=in}}^{{out - 1}} {\frac{{1.2}}{{360}} \times \pi \times (r_{{i,j+1}}^{2}} - r_{{i,j}}^{2})} \times \frac{1}{2} \times (d_{{i,j+1}}^{\prime }+d_{{i,j}}^{\prime })$$

$$\begin{gathered} Volum{e_{rc}}=Volum{e_{after}} - Volum{e_{before}} \hfill \\ {\text{ }}=\sum\limits_{{i=1}}^{{300}} {\sum\limits_{{j=in}}^{{out - 1}} {\frac{{1.2}}{{360}} \times \pi \times (r_{{i,j+1}}^{2}} - r_{{i,j}}^{2})} \times \frac{1}{2} \times ({d_{i,j+1}}+{d_{i,j}} - d_{{i,j+1}}^{\prime } - d_{{i,j}}^{\prime }) \hfill \\ \end{gathered}$$

Where $d_{{i,j+1}}^{\prime },d_{{i,j}}^{\prime }$ were the tangential values before treatment at positions ${r_{i,j+1}},{r_{i,j}}$, respectively. These values were obtained through data fitting.

We chose $Volum{e_{rc}}$ as the volume data.

#### Correlation of corneal data with AL elongation

With the data obtained in step 7, the relationship between these variables and AL elongation were analyzed using SPSS software.

### Statistical analysis

The distribution of continuous variables was expressed as the mean and standard deviation, while categorical variables were presented as the numbers and percentages. A paired *t*-test was used to test the difference in the AL distribution between the baseline and 1-year follow-up. The relationship of the height, area, and volume of the CRC region with the equivalent spherical error, and the relationship of AL elongation with the height, area, and volume of the CRC region were preliminarily explored through scatter diagrams and regression lines based on least squares fitting. A linear regression model was constructed to investigate the relationship of AL elongation with the height, area, and volume of the CRC region after adjusting for confounding variables. The fitting effect of the model was assessed by adjusting the R-square, and the optimal model was selected. Finally, we further explored the relationship of AL growth with the area and volume of the CRC region under different degrees of myopia. All statistical analyses were performed using SPSS software (*v*26.0; SPSS Inc., Chicago, IL, USA) with the significance level established at two-sided *P* < 0.05.

### Patient and public involvement

Patients or the public were involved in the design, conduct, reporting, and dissemination plans of our research.

## Results

### Demographic characteristics

The current study consecutively recruited 77 eligible patients treated with Ortho-K lenses between June 2019 and September 2021, and the relevant data at the baseline and 1-year follow-up were collected. The age of the included patients ranged from 8 to 14 years, with an average of 10.13 ± 1.70 years. There were 32 (42.6%) boys and 45 (58.4%) girls. The SE of the right eye of the included patients ranged from − 0.75 D to -5.50 D, with an average of -3.27 ± 1.29 D. The flat K and steep K values were 43.06 ± 1.37 and 44.16 ± 1.48, respectively. The flat E value was 0.62 ± 0.11, and the pupil size was 5.62 ± 1.36 mm. The AL of the right eye was 24.72 ± 0.83 mm at the baseline and 24.92 ± 0.84 mm 1 year later; thus, the AL increased by 0.19 ± 0.14 mm (*P* < 0.05).

### Correlations among the height, area, and volume of the corneal refractive change region

The amount of CRC was calculated based on the corneal topography maps after 3 months of Ortho-K lens wear. The average height of the CRC region was 9.77 ± 0.60 D; the average area was 16.66 ± 3.61 mm^2^, and the average volume was 87.47 ± 8.96 D*mm^2^.

The height, area, and volume of the CRC region were all correlated with each other. The Pearson correlation coefficient was 0.271 between the height and area, 0.528 between the height and volume, and 0.538 between the area and volume.

### Relationship of AL elongation with the height, area, and volume of the corneal refractive change region

**Fig. 2:**
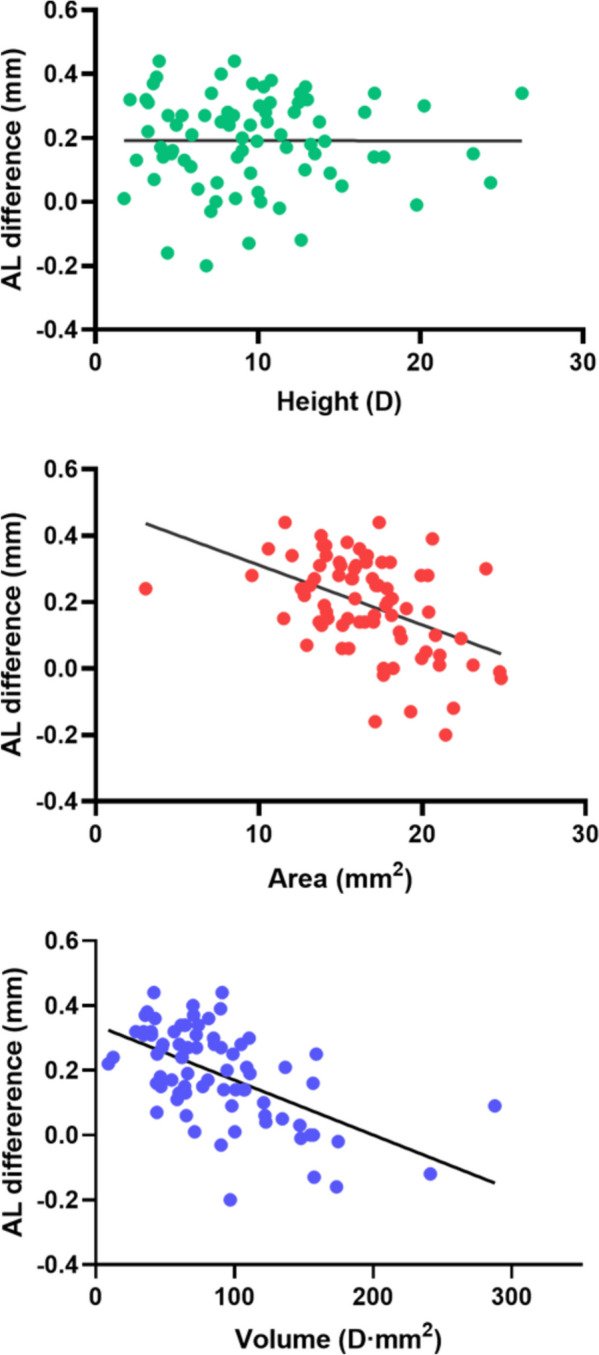
Scatter diagrams showing the relationship of AL elongation with the height, area, and volume of the corneal refractive change region.  AL, axial elongation

At the 1-year follow-up after Ortho-K lens wear, the average AL elongation relative to the baseline was 0.19 ± 0.14 mm. Univariate linear regression analysis showed no significant linear relationship between AL elongation and the height of the CRC region (AL = -0.00005 × height [D] + 0.1919, *P* = 0.9859). For every 1-mm^2^ increase in the area of the CRC region, the average AL elongation decreased by 0.018 mm (AL = -0.018 × area [mm^2^] + 0.4915, *P* < 0.0001). For every 1-D*mm^2^ increase in the volume of the CRC region, the average AL elongation decreased by 0.0017 mm (AL = -0.0017 × CRC-region volume [D*mm^2^] + 0.3395, *P* < 0.0001; Fig. 2).

### Multivariate analysis of AL elongation and the area and volume of the corneal refractive change region

The multivariate model incorporated factors such as the area, volume, gender, age, baseline AL, corneal thickness, flat K, flat E, and pupil size. We found that for every 1-mm^2^ increase in the area of the CRC region, the average AL elongation decreased by 0.01 mm (95% confidence interval (CI): − 0.019 to -0.002, *P* = 0.019). For every 1-D*mm^2^ increase in the volume of the CRC region, the AL elongation decreased by an average of 0.002 mm during the 1-year follow-up (95% CI: − 0.002 to -0.001, *P* < 0.001; Table 1). The adjusted R-squared of the model was 0.485, and the collinearity diagnostics showed no significant collinearity.

**Table 1:** Correlation of AL elongation with the area and volume of the corneal refractive change region

Variable	β	Standard error	Standard β	95% CI	*P* value
Intercept	-1.843	1.130		(-4.105, 0.419)	0.108
CRC-region area	-0.010	0.004	-0.260	(-0.019, -0.002)	0.018
CRC-region volume	-0.002	0.000	-0.518	(-0.002, -0.001)	< 0.001
Gender					
Male	0.040	0.030	0.137	(-0.021, 0.102)	0.190
Female (ref.)					
Age	-0.025	0.008	-0.284	(-0.041, -0.008)	0.004
Baseline AL	0.061	0.025	0.362	(0.011, 0.111)	0.018
Corneal thickness	0.000	0.000	0.088	(0.000, 0.001)	0.354
Flat K	0.016	0.014	0.149	(-0.012, 0.043)	0.253
Flat E	0.201	0.127	0.151	(-0.052, 0.454)	0.117
Pupil size	0.011	0.011	0.090	(-0.011, 0.034)	0.320

The adjusted R-squared of the model was 0.485

*AL *Axial length, *CI *Confidence interval

### Relationship of SE with the height, area, and volume of the corneal refractive change region

We found a significant negative correlation of SE with the height (*P* < 0.0001) and volume (*P* = 0.0017) of the CRC region. The correlation between SE and the area of the CRC region was not significant (*P* = 0.060).

We divided the patients into 2 subgroups based on SE. In the SE ≤ -3.0 D subgroup (SE ≤ -3.0 D means a myopic refractive error > 3.0 D), no significant correlation was found between the area of the CRC region and AL elongation (*P* = 0.555) after adjustments for age, gender, baseline AL, corneal thickness, flat K, flat E, and pupil size. For every 1-D*mm^2^ increase in the volume of the CRC region, the average AL elongation decreased by 0.002 mm (95% CI: − 0.003 to -0.0003, *P* = 0.018). The adjusted R-squared value was 0.506 (Table 2).

**Table 2:** Correlation of AL elongation with the area and volume of the corneal refractive change region in the SE ≤ -3.0 D subgroup

Variable	β	Standard error	Standard β	95% CI	*P* value
Intercept	-3.392	1.680		(-6.954, 0.170)	0.061
CRC-region area	-0.004	0.006	-0.110	(-0.016, 0.009)	0.555
CRC-region volume	-0.002	0.001	-0.464	(-0.003, -0.0003)	0.018
Gender					
Male	0.033	0.049	0.134	(-0.071, 0.136)	0.511
Female (ref.)					
Age	-0.021	0.013	-0.243	(-0.049, 0.007)	0.136
Baseline AL	0.101	0.048	0.645	(-0.001, 0.203)	0.052
Corneal thickness	0.000	0.001	0.044	(-0.001, 0.002)	0.812
Flat K	0.027	0.018	0.341	(-0.012, 0.066)	0.162
Flat E	0.212	0.221	0.155	(-0.256, 0.680)	0.351
Pupil size	0.026	0.018	0.236	(-0.012, 0.064)	0.162

The adjusted R-squared was 0.506

*AL* axial length, *SE* spherical equivalent, *CI* confidence interval

In the SE > -3.0 D subgroup, the volume of the CRC region was not significantly correlated with AL elongation, after adjustments for age, gender, baseline AL, corneal thickness, flat K, flat E, and pupil size (*P* = 0.101). For every 1-mm^2^ increase in the area of the CRC region, the AL elongation decreased by 0.017 mm (95% CI: − 0.030 to -0.003, *P* = 0.016). The adjusted R-squared was 0.496 (Table 3).

**Table 3:** Correlation of AL elongation with the area and volume of the corneal refractive change region in the SE > -3.0 D subgroup

Variable	β	Standard error	Standard β	95% CI	*P* value
Intercept	-4.355	2.323		(-9.088, 0.378)	0.070
CRC-region area	-0.017	0.007	-0.390	(-0.030, -0.003)	0.016
CRC-region volume	-0.001	0.001	-0.310	(-0.002, 0.0002)	0.101
Gender					
Male	-0.012	0.044	-0.040	(-0.101, 0.077)	0.779
Female (ref.)					
Age	-0.034	0.011	-0.400	(-0.056, -0.011)	0.005
Baseline AL	0.096	0.045	0.553	(0.004, 0.188)	0.041
Corneal thickness	0.001	0.001	0.157	(-0.0004, 0.002)	0.226
Flat K	0.055	0.027	0.439	(-0.001, 0.111)	0.055
Flat E	0.183	0.166	0.139	(-0.155, 0.521)	0.279
Pupil size	0.002	0.014	0.017	(-0.027, 0.032)	0.881

The adjusted R-squared was 0.496

*AL *Axial length, *SE *Spherical equivalent, *CI *Confidence interval

## Discussion

The mechanism underlying the myopia-control effect of Ortho-K lenses may be attributed to a mechanism of changing the corneal power distribution between the central and paracentral corneal areas. This change provides the myopic stimulation required in the paracentral foveal area, while the central area is still clearly focused [24]. Although many believe that this therapeutic effect is the result of the displacement of peripheral retinal myopia caused by peripheral steepening of the cornea induced by Ortho-K lens wear [25], to the best of our knowledge, there is no direct evidence to confirm this hypothesis.

In children with myopia, the peripheral defocus on the retina varies across different meridians [16], which suggests that the spatial distribution of the relative corneal refractive power [26] may be more significant than the peripheral defocus. Zhong et al. [21] used the summed corneal power shift within the 7.2-mm chord to predict AL elongation, and found its predictability to be good. However, since the area of the peripheral cornea is larger than that of the central cornea, the refractive change in the peripheral area may constitute a greater influencing factor. Therefore, there may be some limitations to the application of the above parameter [22]. Based on the theory of the areal summation effect proposed by Wallman and Winawer [14], the current study applied an algorithm to quantify the data of the total area, height, and volume of the CRC region formed due to the change in corneal refractive power after Ortho-K lens wear.

The results of the multivariate model showed that AL elongation after wearing Ortho-K lenses for 1 year was negatively associated with the area and volume of the CRC region, but not with the height. The larger the area and volume of the CRC region formed after Ortho-K lens wear, the slower the AL elongation. The relationship between the volume of the CRC region and AL-growth control is consistent with the finding reported by Hu et al. [22] that a curved, pointed pan-like defocus ring was associated with a better myopia-control effect than a flat pan-like defocus ring. The current study took 3 parameters of the CRC region into consideration to provide further quantified data and expound on the possible correlation between defocus and AL change from 3 aspects. The results were consistent with the result reported by Hu et al. The current study did not find the height of the CRC region to be associated with the myopia-control effect; however, the volume and area of the CRC region were correlated with AL growth. Hence, the area and volume of the CRC region might be important indicators for the prevention and control of myopia. Although a correlation between height and myopia control was not identified in the current study, this could be due to the study limitations. The follow-up duration was 1 year, which might not be long enough for a correlation to be observed. In addition, the sample size may have failed to ensure sufficient statistical power to identify this correlation. The results might differ if the follow-up duration was longer. Therefore, caution should be taken when interpreting the correlation between CRC-region height and AL growth.

We found a significant negative correlation between the SE and the height (*P* = 0.0021) and volume (*P* = 0.0017) of the defocus ring. The correlation between SE and the area of the CRC region was not significant (*P* = 0.0607), but the obtained P value was relatively small. Therefore, this result may also differ with a larger sample size. To further observe the correlation between AL control and the amount of CRC in patients with different degrees (mild or moderate) of myopia, we divided the patients into 2 subgroups.

We found a negative correlation between the area of the CRC region and AL elongation in the SE > -3.0 D subgroup. When the area of the CRC region increased by 1 mm^2^, the annual AL elongation decreased by 0.017 mm, which suggested that in patients with severe myopia, the area of the CRC region at the baseline would be large, and thus, the myopia-control effect would be better. This finding is consistent with previous results, namely, the higher the degree of myopia at the baseline, the better the myopia-control effect of Ortho-K lenses [27–29]. In addition, the results of the subgroup analysis showed that the myopia-control effect of Ortho-K lenses was related to the amount of CRC, as speculated by other researchers [21, 30]. Generally, when using Ortho-K lenses for the correction of relatively severe myopia, lenses with flat base curves should be used to make the cornea more oblate (the central area would be flat, and the power toward the peripheral area would increase), which may reduce peripheral hyperopic defocusing, and thereby have a greater inhibitory effect on AL elongation [28]. For patients with SE ≤ -3 D, the volume of the CRC region was negatively correlated with AL elongation. Although we stratified the patients by SE to observe the correlations between AL control and the amount of CRC in different degrees (mild or moderate) of myopia, the possibility that the small sample size did not provide sufficient statistical power to identify a correlation cannot be ruled out. Nevertheless, the correlation between mild myopia and the CRC-region volume and between moderate myopia and the CRC-region area suggest that it is important to consider different parameters when prescribing Ortho-K lenses for adolescents with different degrees of myopia.

The above findings also show that the amount of CRC and myopia-control effect varied with SE, indicating that the volume and area of the CRC region should be taken into consideration when assessing the AL-control effect of Ortho-K lenses. Additionally, the multivariate model showed that the baseline AL and the AL elongation at 1 year were positively correlated, indicating that patients with a longer baseline AL may experience faster AL growth. Therefore, for patients with relatively severe myopia, intervention should be carried out as soon as possible.

Myopia prevention and control is a very complicated issue, as it is difficult to obtain accurate data on personal behaviors and habits such as the duration of outdoor activities and the intensity of near/distance work. The data used in the current study provide an objective description of the correlation between Ortho-K lens wear and myopia control.

Chen et al. [31] studied the effect of pupil diameter on AL growth in Chinese children during Ortho-K treatment, and found that the larger the pupil size, the slower the axial growth. They speculated that the greater the intensity of myopic defocus resulting from a larger pupil diameter may have an inhibitory effect on axial growth. Hence, they concluded that a larger pupil diameter would be conducive to slowing the axial growth, presumably due to the increased peripheral myopic defocus. In the current study, pupil size was not measured in a dark room; rather, it was collected with the IOLmaster under normal indoor light. Therefore, one of the limitations of the current study is that external research conditions were assumed to be the same, but the pupil size (specifically, the optical zone) may influence the amount of myopic defocus after Ortho-K lens wear, thereby affecting the myopia-control effect. In our next study, we will investigate factors affecting the pupil size.

Although Ortho-K lenses can slow down myopia progression, their effect varies greatly among individuals, and thus, it is crucial to identify the patient group in which Ortho-K lenses are most effective. To develop effective strategies to control the development and progression of myopia, it is necessary to clearly understand the physiological and biological processes involved. At present, the relationship between the amount of peripheral refraction and AL elongation is unclear. From the perspective of defocus theory, this study could provide a new option for the prediction of the clinical effects of Ortho-K lenses on myopia control, and it further supports the defocus theory.

The current study has some limitations. This was a retrospective study, and the sample size was relatively small, which may compromise the statistical power. Therefore, further prospective studies with larger sample sizes are recommended.

## Conclusions

In our study, we found the larger the area and volume of the CRC region formed after Ortho-K lens wear, the better the myopia-control effect, as reflected by the AL elongation. For patients with mild-to-moderate myopia, it is necessary to comprehensively consider the area and volume of the CRC region formed when assessing the expected myopia-control effect. The findings of the current study suggest that according to the myopic defocus theory, an improved design to increase the area and volume of the CRC region after Ortho-K lens wear may contribute to a better myopia-control effect.

### Supplementary Information

**Additional file 1:**  **Figure S1.** Calculation of the corneal refractive change region. **Figure S2.** The planform of the defocus ring. The red region is the defocus ring.

## Data Availability

The datasets used and/or analyzed during the current study are available from the corresponding author on reasonable request.

## Abbreviations

Ortho-K: Orthokeratology
AL: Axial length
SE: Spherical equivalent
