# Supplementary material for: Relationship between myopia control and amount of corneal refractive change after orthokeratology lens treatment
Source: BMC Ophthalmol. 2023 Oct 30;23:439. doi: 10.1186/s12886-023-03178-8 (PMC10617139; doi:10.1186/s12886-023-03178-8)
Supplement: Supplementary file 1 — Additional file 1: Figure S1. Calculation of the corneal refractive change region. Figure S2. The planform of the defocus ring. The red region is the defocus ring. [file 12886_2023_3178_MOESM1_ESM.docx]

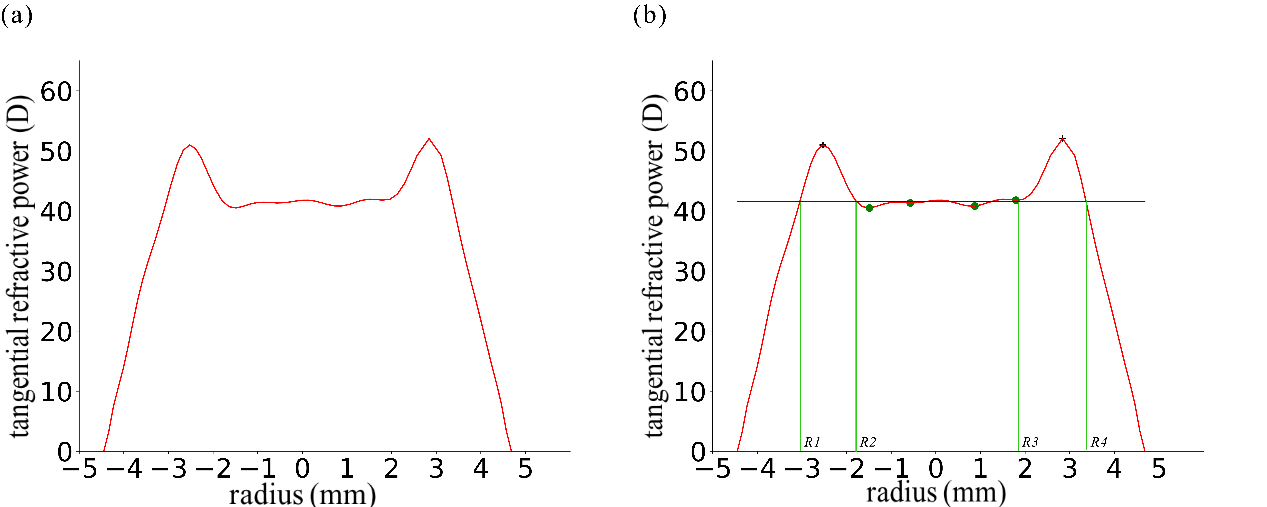


**Figure S1.** **(a)** The results of polynomial fitting. The red line represents the result of fitting. **(b)** Defining the boundary of the defocus ring. + represents the maximum point; · represents the minimum point; the blue line represents the diopter value of the defocus ring boundary; and $R1,R2,R3,and R4$represent the distance values of the defocus ring boundary.


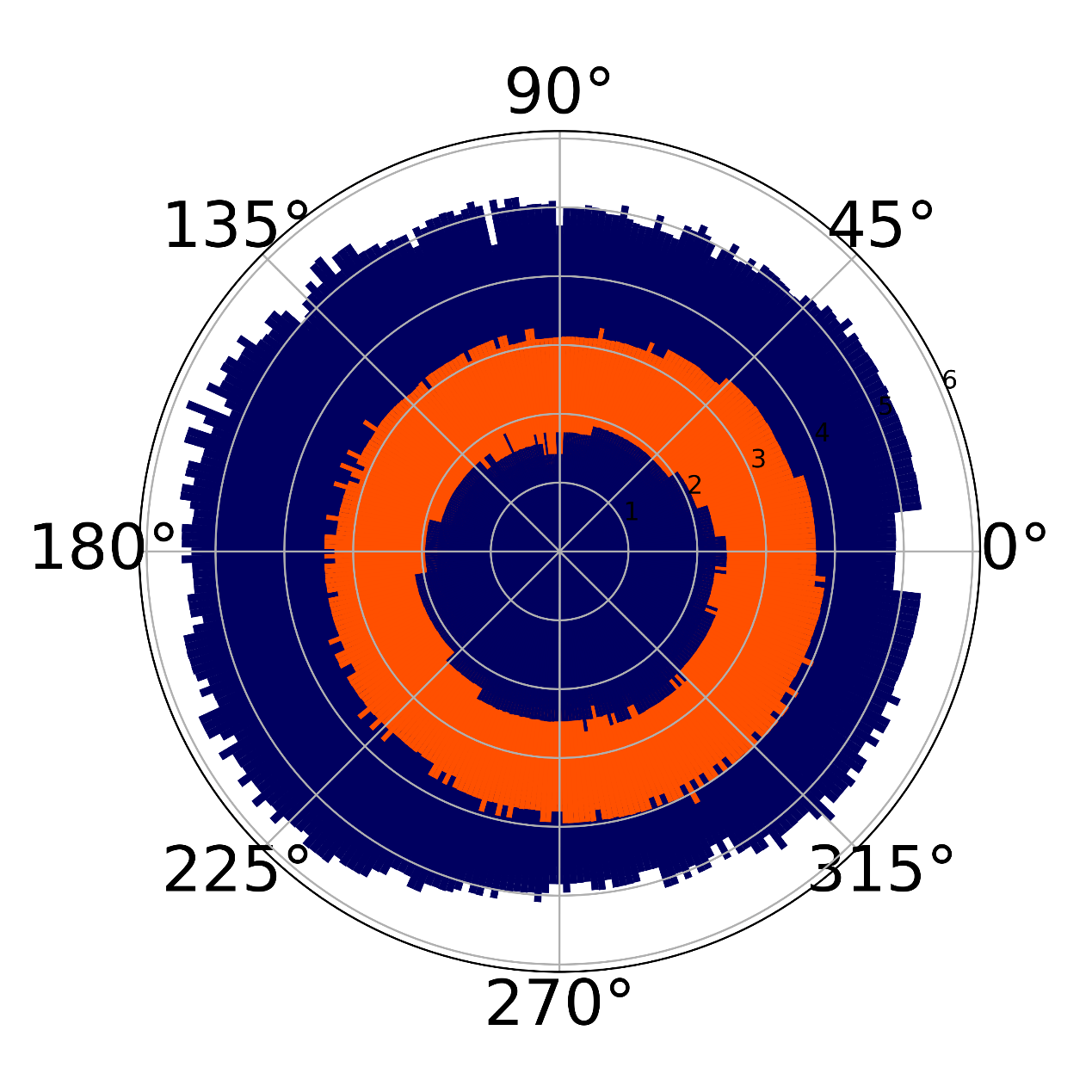


**Figure S2.** The planform of the defocus ring. The red region is the defocus ring.
